# Sampling, Logistics, and Analytics of Urine for RT-qPCR-based Diagnostics

**DOI:** 10.3390/cancers13174381

**Published:** 2021-08-30

**Authors:** Rosel Kretschmer-Kazemi Far, Kirsten Frank, Georg Sczakiel

**Affiliations:** Institut für Molekulare Medizin, Universität zu Lübeck and UKSH, Campus Lübeck, Ratzeburger Allee 160, 23538 Lübeck, Germany; rosel.kretschmerkazemifar@uni-luebeck.de (R.K.-K.F.); kirsten.frank@uni-luebeck.de (K.F.)

**Keywords:** primer design, RNA isolation, RNA stability, RT-qPCR, tumor marker

## Abstract

**Simple Summary:**

Non-invasive tumor diagnosis includes liquid biopsy withdrawn from extracellular and extratissue samples including human urine in which RNA-based markers can be measured by various means. RNA markers include short-chain RNA such as microRNA and long-chain linear and circular RNA. This study describes all steps between sample acquisition, sample stabilization, shipping, and the quantitative determination of RNA-based biomarkers by RT-qPCR that are related to non-coding and coding polymerase II transcripts including mRNA. We aim to provide a novel and thorough easy-to-perform description of all technical and logistics steps of urine RNA-based diagnostics.

**Abstract:**

Body fluids in the context of cancer diagnosis are the primary source of liquid biopsy, i.e., biomarker detection that includes blood and serum, urine, and saliva. RNA represents a particular class of biomarkers because it is thought to monitor the current status of gene expression in humans, in organs, and if present, also in tumors. In case of bladder cancer, we developed a scheme that describes, in detail, all steps from the collection of urine samples from patients, stabilization of samples, their transportation, storage, and marker analysis by qPCR-based technology. We find that urine samples prepared according to this protocol show stability of RNA over more than 10 days at unchilled temperatures during shipping. A specific procedure of primer design and amplicon evaluation allows a specific assignment of PCR products to human genomics and transcriptomics data collections. In summary, we describe a technical option for the robust acquisition of urine samples and the quantitative detection of RNA-based tumor markers in case of bladder cancer patients. This protocol is for general use, and we describe that it works for any RNA-based tumor marker in urine of cancer patients.

## 1. Introduction

In the field of cancer diagnostics, non-invasive technologies are increasingly attractive for initial diagnosis of tumors and for therapy monitoring [1,2,3]. Body fluids are the primary source of liquid biopsy, i.e., biomarker detection, and include blood and serum, urine, and saliva [4,5]. In most body fluids and in urine, in particular, nucleic acids can be found in sub-compartments and in extracellular vesicles that can be isolated including circulating tumor cells (CTC’s), exosome-like particles, ectosome-like particles, and apoptotic bodies [6,7,8,9,10,11]. Among the chemical entities of biomarkers, RNA plays a prominent role because RNA is thought to monitor the current status of gene expression, i.e., the relevant transcriptome. In the case of blood, circulating cell-associated, vesicle-associated, and cell-free RNA seems to reflect the patterns of current gene expression of the organism, individual organs, and include transcripts originating from malignant cell growth [12,13,14,15].

Cell-free nucleic acids including cell-free RNA in urine may originate from cell necrosis, cell apoptosis, active secretion by healthy and by tumor cells of the urinary tract, and transport of circulating RNA from blood into urine [16]. Even though urinary nucleic acids are fragmented, they can be used to detect marker sequences such as microRNAs and they are long enough for PCR-based detection of fragments larger than approximately 100 nucleotides [17]. It is reasonable to assume that the stability of RNA in urine is influenced by RNA structure, packaging together with polypeptides, and cationic and/or lipophilic macromolecules into larger complexes, as well as by nuclease activity in urine.

On the diagnostic clinical level, however, these issues are not dominant, whereas technical parameters directly influence the feasibility of using the composition of urine RNA as a source of strong diagnostic markers. In this article, we describe a series of relevant parameters for the diagnostic chain from sample acquisition to laboratory determination of tumor markers.

## 2. Results

**Figure 1:**
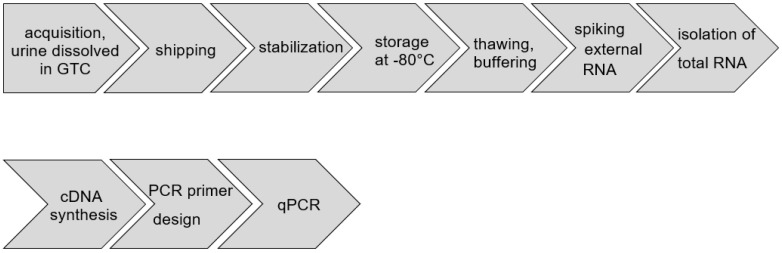
Standardized processes for acquisition, shipping, treatment, and RT-qPCR-based analysis of urine RNA.

In the following, we describe all steps from clinical sampling of urine, sample delivery, sample treatment, isolation of total RNA, synthesis of cDNA, primer design for qPCR, and qPCR (Figure 1). All technical steps are described in detail in the Materials and Methods section. In the following, we provide explanations and control experiments illuminating the conditions and protocols. We also provide arguments for this final version of this diagnostic protocol of laboratory steps.

It is noteworthy that we aim to isolate total RNA, regardless of the sub-fraction in which it is contained. Initial studies on the distribution of RNA in sub-fractions of urine samples indicate that RNA can be found in cell pellets, sediments produced by various g-values, or in solution/supernatant. The amounts of RNA, however, varied substantially within different samples. Thus, in order to exclude influences by cell fractions, we used total urine without further steps, e.g., centrifugation. This is thought to give rise to the analysis of the complete set of urine RNAs.

### 2.1. Clinical Sampling of Urine

**Figure 2:**
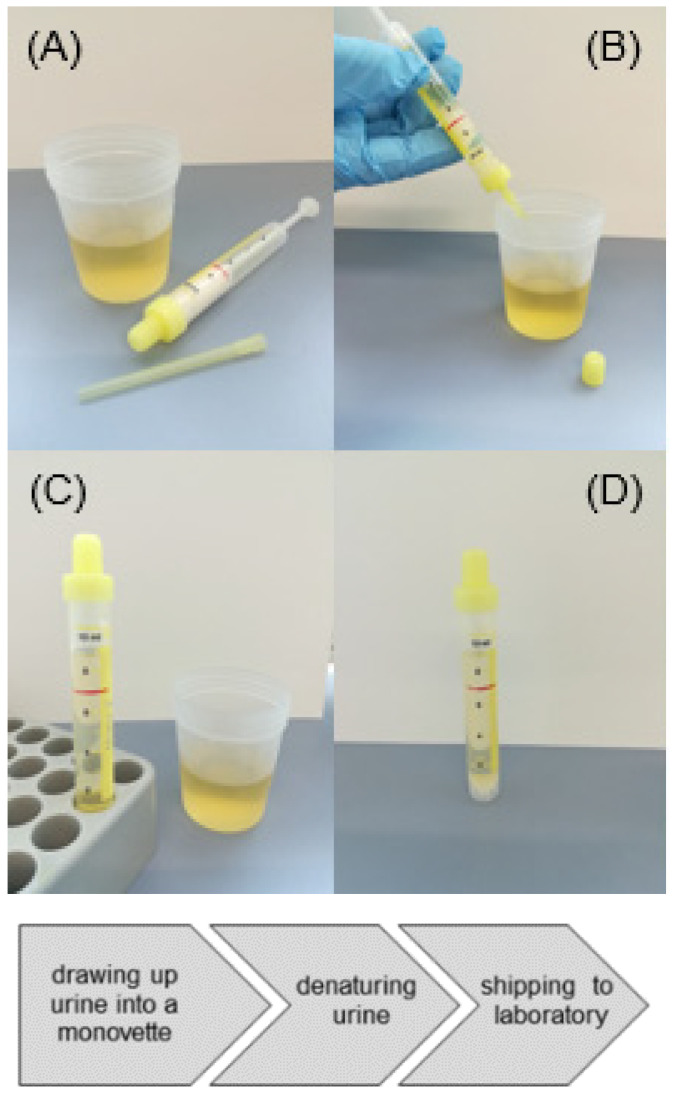
Illustration of soaking a urine sample from a beaker into a monovette (plastic syringe), pre-loaded with GTC (**A**,**B**). The stamp of the plastic syringe is broken off after filling and the sample is ready for shipping (**C**,**D**).

Urine samples were acquired from patients at hospitals or doctor’s offices where donors had been informed about the background and the diagnostic steps, and where they signed a written consent. Approximately 7 mL of urine were drawn up into a monovette, pre-filled with 3.54 g of guanidinium thiocyanate (GTC) (Figure 2). The guanidinium salt was dissolved by gentle shaking of the monovette. The resulting sample volume is 8.33 mL. All steps were performed at room temperature including shipping, a step at which samples might have been exposed to a range of temperatures.

### 2.2. Shipping Urine Samples

**Figure 3:**
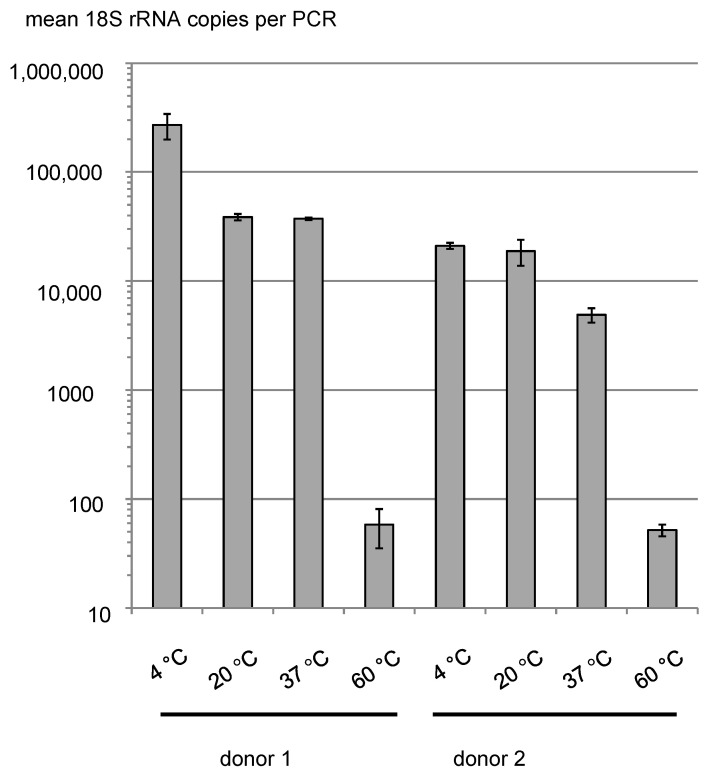
Influence of storage temperature on RNA signal strength during a 3-days period as a measure for shipping.

Once urine samples had been soaked into monovettes containing GTC, the RNA in solution seems to be relatively stable. However, to gain additional insight into the major parameters of RNA stability, we first measured the temperature dependency on the detectability of 18S rRNA for two individual donors. We chose 18S rRNA because this is the most abundant RNA species in urine samples and, thus, it allows a large dynamic range of amounts of RNA to be quantified. Fresh urine samples were dissolved in GTC in monovettes and kept at constant temperatures varying between 4 °C and 60 °C, which we feel, covers a temperature range that is realistic for shipping and storing samples (Figure 3). This test shows a temperature-dependent decrease in detectable 18S rRNA by one order of magnitude between storage at 4 °C and 37 °C. A further increase in the temperature to 60 °C seems to be related to a substantial loss of signal by more than two orders of magnitude. This seems to be a loss that could be too great in the case of RNA species occurring at very low amounts in urine. Conversely, losses below a factor of ten seem to be tolerable when considering RT-qPCR as the method for detection.

However, we wish to stress that this methodology has not been tested for shipping temperatures above 37 °C. Thus, for very warm countries or summer seasons we recommend to carefully consider or control shipping temperatures.

**Figure 4:**
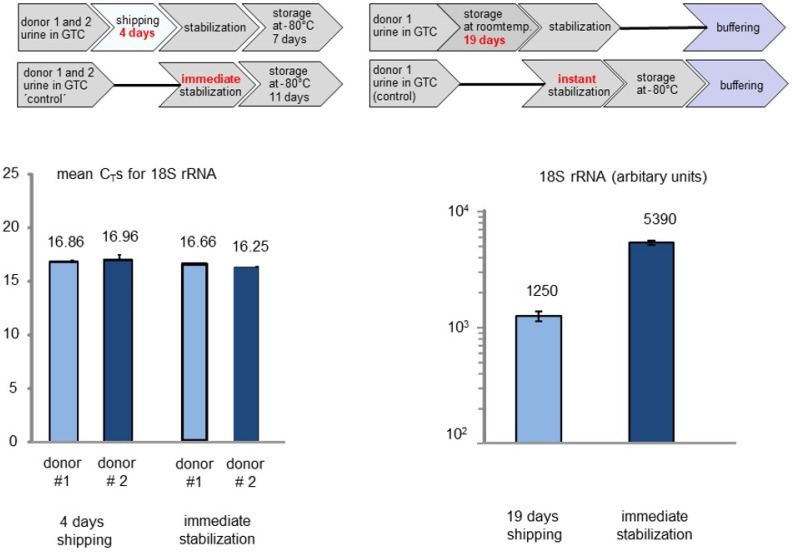
Influence of 4-days and 19-days storage at room temperature or shipment temperature, respectively, on the detectability of 18S rRNA. We included two donors, performed two independent RNA isolations for each donor and two RT-qPCR reactions per RNA preparation. After storage at −80 °C all samples were buffered which is indicated for the 19-days data (upper right panel).

The second most critical parameter related to RNA stability in urine samples seems to be related to the time period of shipment or storage at room temperature. In order to shed light on this factor, we shipped monovettes containing urine samples as a regular postal parcel, which took 4 days until arrival. In addition, we kept monovettes containing urine samples for a 19-days period at room temperature before the stabilization step (Figure 4). The control samples were stored at −80 °C before RNA was prepared and 18S rRNA was detected, as described. We included two donors, performed two independent RNA isolations for each donor and two RT-qPCR reactions per RNA preparation.

In summary, the time period between sample acquisition and storage of samples at −80 °C does not seem to be related to a major loss of detectable amounts of RNA. A stability study was performed with 18S rRNA (Figure 3 and Figure 4). In this experiment, the amounts of RNA remain almost stable even after 4 days of commercial standard transport without cooling.

The temperature during shipment seems to have a minor influence on RNA stability below 37 °C while the 60 °C values indicate degradation at higher temperatures (Figure 3). Under real conditions of shipping, the temperature limit of 37 °C does not seem to be exceeded, which is compatible with the stable measurements of RNA content of a great number of shipped samples from hospitals.

### 2.3. Laboratory Handling, Storage, and Thawing Samples

After arrival of samples and before freezing them, the pH value was adjusted to 7.0 by addition of 500 μL of 1 M sodium acetate buffer pH 7.0 (final concentration of 56 mM), and the addition of 167 μL of 3% N-lauroylsarcosine. This resulted in a total volume of 9 mL. After gentle mixing, samples were shock-frozen in liquid nitrogen and stored at −80 °C. For taking aliquots, e.g., 2.25 mL, samples were thawed at room temperature under mild shaking and 250 µL of 1 M HEPES buffer pH 7.0 was added. The final concentrations in the urine sample were 3 M GTC, 50 mM sodium acetate, 0.5% N-lauroylsarcosine, and 100 mM HEPES, pH 7.0.

Regarding the storage temperature of −80 °C, it should be noted that samples were stored for many years. It turned out that the highest signal stability, i.e., RNA stability was achieved by storing at this temperature. We would like to emphasize that we mean ‘functional stability’ rather than ‘physical stability’, i.e., the read-out is signal strength in RT-qPCR tests. However, we are not aware of data describing physical RNA stability in fractions of urine including different extracellular vesicles and, in particular, not in the presence of GTC. More specific information on the storage temperature cannot be provided but we feel that the storage temperature gives rise to robust and reproducible marker data.

Further, we usually froze urine samples at −80 °C after sub-division into aliquots such that aliquots were thawed only once for preparation of total RNA and freeze-and-thaw cycles were avoided.

### 2.4. RNA Isolation and cDNA Synthesis

The origin of RNA in urine samples is thought to contain a number of sources including living or non-viable cells, cell surface-bound RNA [18,19], naked RNA, and RNA associated with or contained in various kinds of extracellular vesicles [20]. Although it is reasonable to assume that the high concentration of GTC in urine samples lyses cells and vesicles, we cannot quantitatively determine the contribution of each of these sources to total amounts of RNA to be monitored by an RT-qPCR-based readout.

Spiking of urine samples with in vitro transcribed RNAs is a part of quality assurance. Here, we used a standard luciferase-derived in vitro transcript, which is added at defined amounts (10^7^ copies) to urine samples after buffering with HEPES pH 7.0 and before the RNA isolation is started in the use of a commercial RNeasy Midi Kit.

We feel that it is noteworthy that the extracellular environment, including blood and, very likely, also urine, contain a mixture of ribonucleases. While GTC will denature most of them, it remains open to some extent whether there is still remaining degradation of RNA in a way that is different between various sources of RNA in urine.

In summary, we describe a robust and reproducible protocol for the isolation of total RNA from urine, which has been described in detail [21]. This protocol had been working for the detection of micro RNA [22] and for long-chain RNA [21].

The synthesis of cDNA was performed according to standard protocols (Section 3).

### 2.5. Primer Design for PCR

Convergent primers for qPCR-based detection of RNA species that serve as potential diagnostic candidates and TaqMan probes were designed according to the following steps.

First, we selected target transcripts by checking the NCBI Reference Sequence (RefSeq) Database. Here, we searched for annotated, non-redundant sequences for DNA, RNA, and proteins including a platform for sequence data with genetic and functional information.

Subsequently, we used the RefSeq data to define a set of target RNA sequences and considered all known transcript variants (‘NM_XXXXX’). Further, we considered non-translated sense transcripts, antisense transcripts, and pseudogene-derived sequences (‘NR_XXXXX’) whereas we did not consider SNPs.

A selection of target-specific primers was derived from NCBI Primer-BLAST and Primer3 algorithms. In addition, we used BLAST and global alignment algorithms (‘user selected’). The tool ‘Primer3 version 4.0.0’ was used to consider and to analyze criteria including T_m_, length, GC content, potential of primer dimers, total length of amplicons, and specificity. The use of TaqMan probes and their design were included only in case of failure of Primer-Blast. This can be supported by tools provided by commercial suppliers of TaqMan probes (e.g., Metabion, Planegg/Steinkirchen, Germany).

**Figure 5:**
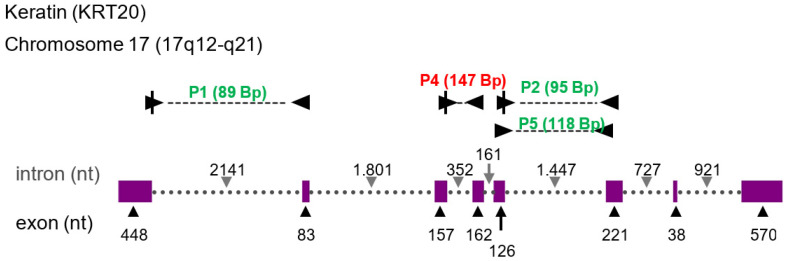
Depiction of selection criteria for diagnostic amplicons for PCR-based marker detection. This example shows the transcription map of the keratin (KRT20) locus. The upper panel depicts schematically the KRT20 transcript. Exons are indicated by pink bars, dashed lines indicate introns. Filled arrow heads with a vertical line indicate exon-exon spanning primers. The length of exons and introns are indicted along the transcript line. Primers and their amplicons are depicted by triangles and a dashed line in between. The amplicon length is indicated by numbers of nucleotides in green color. The amplicon indicated by red color has not full filled the criteria for specificity and/or sensitivity.

The final selection of primers included the following settings. T_m_ 57–63 °C (optimal T_m_, 60 °C). Data bank for *Homo sapiens*, RefSeq mRNA (“NM”) or RefSeq RNA („NR“). As far as possible, all primer pairs should be specific for all transcript variants of a given locus (Figure 5). Either of the forward or reverse primer spans an exon-exon fusion site at a minimal length of 7 nucleotides at the 5′-end of exons and 4 nucleotides of the 3′-end of exons. Primers need to span at least one intron. The length of all amplicons was adjusted to a length between 70 and 150 nucleotides.

After the selection of amplicons, all primers are tested for purity, quantity, and size by UV absorption measurements and by gel electrophoresis.

### 2.6. qPCR-Based Detection of RNA Sequences

**Figure 6:**
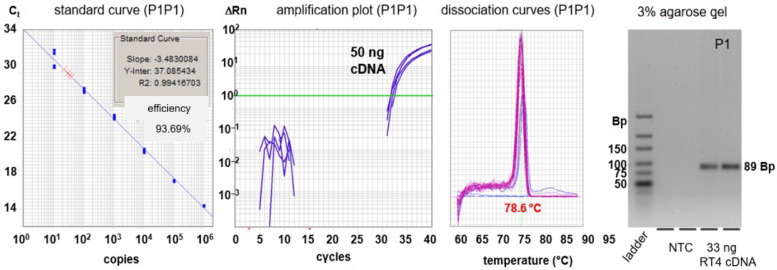
Experimental evaluation of selected PCR amplicons, i.e., primer pair P1 (see Figure 5). Left panel, standard dilution curve of cloned KRT20 (plasmid pCRII-KRT20P1P1); middle left panel, amplification plots of cDNA prepared from RT-4 cells; middle right panel, dissociation curve of the amplification product; right panel, gel analysis of the amplification product that shows the calculated size of 89 base pairs (abbreviation: NTC: non template control).

Prior to diagnostic PCR reactions, we checked the specificity and sensitivity of primer pairs by dilution series of standards and evaluation of the products of PCR by dissociation measurement and gel electrophoresis (Figure 6). All primer pairs that did not full fill these criteria were excluded.

### 2.7. Marker Ratio—Evaluation of Data

The procedure described in this article results in qPCR-based quantification of potential RNA markers in a diagnostic setting (Table 1). It is important to note that this readout for a single RNA sequence is very difficult to standardize because no measurable reference value is available due to extremely low RNA concentrations in urine samples. Even the most abundant RNA species, i.e., 18S rRNA, varies substantially among individuals and samples.

To solve the problem of reference values, we suggest considering ratios of markers that show an inverse dependency of the health state. For example, in case of bladder cancer (BC) we search for a marker that increases during tumor development and a second marker that decreases during tumor development. The ratio of both has at least two conceptional advantages. Firstly, it is independent of the total amount of urine RNA to be analyzed. Thus, standardization is not necessary. Secondly, the ratio between the healthy state and the disease state seems to be able to indicate a larger dynamic range than single individual marker values.

## 3. Materials and Methods

### 3.1. RNA Isolation

For isolation of urine RNA, a 2.25 mL aliquot of denatured and stabilized urine was thawed with gentle shaking, mixed with 0.25 mL of 1 M HEPES, pH 7.0, and spiked with 10^7^ copies of luciferase RNA transcribed in vitro. RNA isolation was then performed using the Midiprep Kit (Qiagen, Hilden, Germany) according to company instructions. For this, an equivalent volume of 70% ethanol and 25 µL of ß-mercaptoethanol was added to the 2.5 mL sample. 4 mL was added to the column. Subsequent steps were performed following the manufacturer’s instructions, including an on-column hydrolysis of genomic DNA by treatment with DNase I. RNA was eluted twice with 160 μL H_2_O, lyophilized, and resuspended in 16 µL of RNase-free water. Samples were stored at −80 °C.

### 3.2. cDNA Synthesis

Reverse transcription was performed using the SuperScript III First-Strand Synthesis Kit (Thermo Fisher Scientific/Invitrogen, Waltham, MA, USA) in a total volume of 20 µL containing 7.5 µL RNA extract and 300 ng of random hexamer primer (Thermo Fisher Scientific/Invitrogen, Waltham, MA, USA). For Non-RT control reactions, nuclease-free water was added instead of solutions of RNaseOut and reverse transcriptase.

### 3.3. Quantitative PCR (qPCR)

qPCR was performed using the SYBR Green and TaqMan Systems in a total reaction volume of 10 μL in 384-well plates. SYBR Green reactions were performed with SYBR Select Master Mix, with uracil-DNA glycosylase (UDG) (Thermo Fisher Scientific, Waltham, MA, USA). Template volume was 4 µL of 1:16 diluted cDNA or 4 µL of 1:160 diluted cDNA for detection of 18S rRNA. The concentration for each primer was 200 nM. Quantitative PCR was carried out in an ABI PRISM^®^ 7900HT Sequence Detection System (Thermo Fisher Scientific/Applied Biosystems, Waltham, MA, USA) with 50 °C for 120 s (UDG activation), 95 °C for 120 s (hot start), followed by 40 cycles consisting of 95 °C for 15 s, and 60 °C for 60 s. Melting curve analysis was performed. For the TaqMan system, TaqMan Universal Master Mix II, with uracil-N-glycosylase (UNG) (Thermo Fisher Scientific, Waltham, MA, USA) was used. The template volume was the same as for the SYBR Green system. The primer pair concentration and TaqMan probe concentration were 200 nM, and 250 nM, respectively. The thermocycler conditions were 50 °C for 120 s, 95 °C for 600 s, and 40 cycles consisting of 95 °C for 15 s and 60 °C for 60 s. Samples were measured in quadruplicates and negative controls without reverse transcriptase and RNaseOut (Non-RT) or without template (NTC) were included. Standard curves were obtained after amplification of 10^1^ to 10^7^ copies of purified standard plasmids. Data analysis was performed via the SDS 2.4.1 software (Thermo Fisher Scientific/Applied Biosystems, Waltham, MA, USA).

Gene-specific primer sequences, amplicon length, cloned reference sequences, and TaqMan probes are listed in Table 2 and Table 3. 

## 4. Discussion

This study focuses on technical aspects of the acquisition, shipping, and analyses of urine and urine RNA, respectively. The data summarized here describe a standardized methodology for the search of non-invasive urine RNA-based tumor markers. Please note that this study does not describe validated RNA markers for BC. It rather describes the feasibility of this approach. This methodology has been applied in many studies describing RNA-based tumor makers, including microRNA and RNA polymerase II transcripts in human cells [21,22,23,24,25]. In the light of these successful applications of the protocol, this work describes a robust and quantitative technology for RNA-based marker detection in urine samples for diagnostic purposes.

In order to illustrate the usefulness of the protocols, we included qPCR-based results in the use of urine RNA samples in Table 1. The data shown in this table indicate that qPCR signals are measurable and significantly above background. Furthermore, some of the values for certain gene markers differ substantially. We feel that reproducibility, error ranges and, hence, significance of results are worth for further diagnostic studies, including BC. This may include initial diagnoses and the monitoring of patients after therapeutic treatment of BC [26,27].

The examples of marker detection shown in Figure 6, in particular KRT20, seem to reach the gold standard level of the diagnosis of BC. This has warranted a transcriptome analysis of urine RNA obtained from BCa patients and healthy donors [25] from which we currently derive new markers for BCa with increased sensitivity and specificity.

However, completely different settings without isolating total RNA could be considered for optimization of the power of RNA-based markers. This might include the search for compartments that primarily contain specific RNA markers [28]. For example, a more significant relation between the detection of markers and disease might be achieved if specific extracellular vesicles are prepared before RNA is isolated [29,30]. Initial experiments with ectosome-like particles, exosome-like particles and apoptotic bodies isolated from two donors support the possibility of this assumption. It is important to note that in the case of enriching sub-fractions of urine, detergents such as GTC have to be avoided. On the negative side, this might hamper fast and simple acquisition of urine samples. Moreover, in clinical settings and prior to shipping, protocols would become complex without initial stabilization of RNA.

Although RNA is satisfactorily stabilized by GTC, its protection could conceivably be increased by analyzing the role of detergent and its concentration. Similarly, the influence of temperature peaks on amounts and integrity of RNA could be characterized with more detail in order to identify further improvements.

## 5. Conclusions

The acquisition and shipping of urine samples from clinics and doctor’s offices can be organized such that monovettes, pre-filled with GTC, can be safely and stably stored at the place of sample collection, instructions for sampling are available and parcels can even be prepared. Since the shipping step seems to be established as well, we describe a robust procedure prior to storage of samples and laboratory analyses, where one is able to detect RNA markers at low copy numbers of equal to or less than 100 copies per qPCR reaction. This is supported by a detailed and defined scheme of primer design for PCR (Figure 5 and Figure 6).

It is conceivable that many kinds of RNA species and DNA species can be measured in a diagnostic setting by methodology based on this work. This may include long-chain RNA and short RNA strands, e.g., microRNA and circular RNA.

## Figures and Tables

**Table 1:** Mean copy numbers of gene-specific urine RNAs (upper horizontal line) and classification of patient groups (left column). The data set is based on the technology described in this study and indicates potential urine RNA-based markers for BC. For each urine sample, a 4-fold determination was performed by qPCR.

Health Status	KRT20	LASP1	OP18	UPK1A	BIRC5	18S rRNA	Luciferase	No. of Patients
A1	282	1.946	193	406	9	616,126	1001	41
A2	138	410	38	1.206	4	307,401	1349	40
A3	8263	6321	5605	26,517	1022	2,791,914	1852	66
B1	777	18,784	109	530	6	4,300,097	2287	49
B2	12	754	157	1717	21	423,152	1308	4
B3	5	707	5	91	0	72,451	722	3
B4	20	68	6	157	0	39,717	587	12
C	89	328	63	533	6	207,231	1328	47

Abbreviations: A1, low-risk; A2, intermediate-risk; A3, high-risk; B1, urinary tract infection; B2, urolithiasis, B3, relevant malignant co-disease (e.g., prostate carcinoma), B4, relevant benign co-disease (e.g., papilloma/papillary neoplasia, benign prostatic hyperplasia, prostate adenoma).

**Table 2:** Primer pairs and length of PCR amplicons for genes listed in the first column. The plasmid names of cloned standards are listed in the last column.

Gene	Primer Sequences (5′–3′) Upper Line: Forward Lower Line: Reverse	Amplicon (bp)	Standard Plasmid
BIRC5	GACCACCGCATCTCTACATTCA CAAGTCTGGCTCGTTCTCAGT	117	pCRII-BIRC5P2P1
KRT20	AGCTGCGAAGTCAGATTAAGGA GAAGTCCTCAGCAGCCAGTT	89	pCRII-KRT20P1P1
LASP1	CTCGGAACCATGAACCCCA ATGCCAGAACTTATCCAGACAGT	87	pCRII-LASP1P1
OP18	AAAGACGCAAGTCCCATGAAG AGCTTCCATTTTGTGGGTCAG	146	pCRII-OP18
UPK1A	CGGAAGGCTGACGTGAAGT CGTCATGATTGAGCAAGAATGC	72	pcDNA-UPK1A
Luciferase	GAACATCACGTACGCGGAATAC TTTCACTGCATACGACGATTCTG	152	pCRII-Topo-18S
18S rRNA	CACATCCAAGGAAGGCAGCAG GACTTGCCCTCCAATGGATCC	152	pCRII-Topo-18S

**Table 3:** TaqMan probes for qPCR-based detection of OP18, UPK1A, and Luciferase, respectively.

Gene	Sequences (5′–3′)
OP18	FAM-GCAGCTGGCTGAGAAACGAGAGCA-BHQ-1
UPK1A	FAM-TGGCACATCTGGTCCCATGGA-BHQ-1
Luciferase	FAM-TCGAAATGTCCGTTCGGTTGGCA-BHQ-1

FAM, 6-Carboxyfluorescein; BHQ-1, Black Hole Quencher™.

## Data Availability

All the data relative to this study are presented in the manuscript.
